# Nosocomial outbreak of KPC-2- and NDM-1-producing *Klebsiella pneumoniae* in a neonatal ward: a retrospective study

**DOI:** 10.1186/s12879-016-1870-y

**Published:** 2016-10-12

**Authors:** Jing Yu, Kun Tan, Zhihui Rong, Yue Wang, Zhongju Chen, Xuhui Zhu, Li Wu, Li Tan, Wei Xiong, Ziyong Sun, Ling Chen

**Affiliations:** 1 Department of Clinical Laboratory, Tongji Hospital, Tongji Medical College Huazhong University of Science and Technology, Jiefang Road 1095#, Wuhan, 430000 China; 2 Department of Infection Control, Tongji Hospital, Tongji Medical College Huazhong University of Science and Technology, Jiefang Road 1095#, Wuhan, 430000 China; 3 Department of Pediatrics, Tongji Hospital, Tongji Medical College, Huazhong University of Science and Technology, Jiefang Road 1095#, Wuhan, 430000 China

**Keywords:** Multidrug-Resistant, *Klebsiella pneumoniae*, KPC-2, NDM-1, Nosocomial Infection, Neonate

## Abstract

**Background:**

The spread of resistance to carbapenems among *Enterobacteriaceae* has become a major public health problem in recent years. In this study, we describe an outbreak of *Klebsiella pneumoniae* in the neonatal ward. First, we aimed to study the drug resistance, genetic relatedness, and transmission mechanism of carbapenem-resistant *K. pneumoniae*; second, we implemented infection control measures to contain the outbreak.

**Methods:**

We investigated 27 non-repetitive strains isolated from neonates and five strains cultured from around the neonatal ward. Polymerase chain reaction (PCR), the agar dilution method, and multilocus sequence typing (MLST) were used to analyze the resistance gene(s), antimicrobial susceptibility, and homology, respectively. Health-care personnel education, hand hygiene, outer gown changing, and infected patient isolation were strictly enforced.

**Results:**

Our antimicrobial susceptibility results show that all strains were multidrug-resistant. MLST and PCR results revealed that, in this study, all of the KPC-2-producing strains are Sequence Type (ST) 11 (ST11) (*n* = 22) and all of the NDM-1-producing strains are ST20 (*n* = 4) or ST888 (*n* = 1). The environmental strains were identified as KPC-2-positive *K. pneumoniae* ST11 (*n* = 3) and NDM-1-positive *K. pneumoniae* ST20 (*n* = 2). The percentages of isolates with the extended-spectrum-β-lactamases CTX-M-15, blaCTX-M-14, blaTEM-1 were 9.4, 84.3, and 68.8 %, respectively. AmpC β-lactamase genes were not detected in our isolates.

**Conclusions:**

KPC-2-positive *K. pneumoniae* ST11 and NDM-1-positive *K. pneumoniae* ST20 were associated with this outbreak. The identification of these isolates in samples from radiant warmers and nurses suggests that hospital cross-transmission played a role in this outbreak. Active infection control measures were effective for controlling this multidrug-resistant *K. pneumoniae* outbreak.

**Electronic supplementary material:**

The online version of this article (doi:10.1186/s12879-016-1870-y) contains supplementary material, which is available to authorized users.

## Background

Carbapenem-resistant *Enterobacteriaceae* (CRE) have increasingly been found to cause nosocomial infections during the past decade; they primarily affect critically ill patients [1]. Carbapenem-resistant *Klebsiella pneumoniae* (CR-KP) is one of the most important types of CRE, and it has spread extensively around the world [2]. Carbapenems are the most effective drug for treating infections caused by *K. pneumoniae* that produce extended-spectrum beta-lactamases (ESBLs). However, with the increase of carbapenem resistance in *K. pneumoniae*, options for treatment have become limited [3].

Carbapenemases, including KPC, GES, SME, NMC, IMI NDM, IMP, VIM, and OXA-48, are the main mechanisms of carbapenem resistance in CRE. Other mechanisms of carbapenem resistance in these bacteria include the absence or decreased expression of outer membrane proteins and mutations in the ampC enzyme or ESBLs [4].

Outbreaks of hospital-associated CRE infections are not rare [5, 6]. In recent years, outbreaks of CRE infections in neonates have been rising and have attracted increasingly more attention [7, 8]. Here, we noted that the frequency of *K. pneumoniae* isolated from newborns in our neonatal ward had dramatically increased compared with that of previous years. Therefore, we collected CR-KP in 2015 from January to November to study the resistance and transmission mechanisms of carbapenem-resistant strains in the neonatal ward.

## Methods

### Setting of the study

A retrospective study was conducted in an 11months period between January 2015and November 2015 in the neonatal ward of Tongji Hospital (TJH). The neonatal ward of TJH receives approximately 1750 admissions per year and it has six rooms (60 beds) for newborns. The 60 % of patients come from maternity ward and 30 % of neonates were outpatients.

### Bacterial isolates collection and identification

Thirty-two carbapenem-resistant *K. pneumoniae* were included in this study. Twenty-seven isolates were collected from different patients. At the same time, according to previous investigation, hospital infection control staff collected environmental samples from high frequency contact areas in neonatal ward in September 8, 2015, including bed railing, stethoscope, personal digital assistant, suction apparatus, venous catheter, radiant warmer, nurses’ hand, thermostat button and ventilator machine. Samplings were accomplished before routine cleaning, 89 environmental samples and 40 samples from nurses’ hands were obtained. All isolated strains were identified by conventional biochemical methods, then were confirmed by PCR amplification of 16S rRNA gene and sequencing [9].

### PCR and DNA sequence analysis of resistance gene

PCR were performed to detect drug-resistance genes, including carbapenemases (*bla*KPC-2, *bla*GES, *bla*IMP-4, *bla*VIM-1, *bla*NDM-1, *bla*OXA-48), common ESBL genes (*bla*CTX-group1, *bla*CTX-group2, *bla*CTX-group8, *bla*CTX-group9, *bla*TEM-1, and *bla*SHV), and ampC genes (MOX, FOX, DHA, CIT, AAC and EBC) [10–12]. The PCR products were sequenced and analyzed using BLAST (http://www.ncbi.nlm.nih.gov/BLAST).

### Susceptibility testing

Antibiotic susceptibility testing was assayed by agar dilution method to determinate the minimum inhibitory concentration (MICs) of ampicillin, ceftazidime, cefotaxime, cefepime, cefuroxime, cefoxitin, gentamicin, levofloxacin, amikacin, ciprofloxacin, aztreonam, imipenem, meropenem, trimethoprim-sulfamethoxazole, piperacillin-tazobactam, tigecycline and colistin. All antibiotics, except tigecycline and colistin, were interpreted according to the standard of the Clinical and Laboratory Standards Institute (CLSI) document M100-S22. For tigecycline and colistin, the European Committee on Antimicrobial Susceptibility Testing (EUCAST) breakpoint was used. *E. coli* ATCC 25922 and *P.aeruginosa* ATCC27853 were used as quality control.

### Multilocus sequence typing (MLST)

Epidemiological relatedness was carried out by multilocus sequence typing (MLST). Multilocus sequence typing (MLST) for clinical *K. pneumoniae* isolates was performed following the methods described previously [13]. The allelic profiles and sequence types (STs) were assigned using online databases (http://www.pasteur.fr/recherche/genopole/PF8/mlst/Kpneumoniae.html).

### Infection control interventions

Strict infection control measures were implemented according to the Guidance for Control of CRE in November, 2015 [14]. Healthcare personnel are familiar with proper hand hygiene technique and wash their hands properly before entering and upon leaving patient rooms. Hand hygiene adherence was monitored by video. Patients who are suspected or confirmed with CRE should be placed in a private room in which staff and visitors are required to don gloves and gowns for entry. Extensive cleaning of shared equipment and the use of disposable materials (when feasible) were implemented.

## Results

### Epidemiological characteristics of *K. pneumoniae* isolates

The molecular epidemiology of carbapenem-resistant *Enterobacteriaceae* was studied from January to November 2015. During this period, There were 1519 patients admitted to hospital,including 590 females (38.9 %) and 929 males (61.1 %). The sex ratio of all patients is similar to infection group (61.1 vs 63.0 %). The average age of the CR-KP infection (32.5 ± 22.7) ws older than all patients (11.2 ± 23.4).27 consecutive CRE were isolated from 27 different patients. The characteristics of the CR-KP isolated in this study are shown in Table 1. Twenty seven of the isolates are from newborns in the neonatal unit, including 17 males and 10 females, with a mean age of 32.5 days (range, 1–85 days). The sources of these specimens were blood (*n* = 9), urine (*n* = 8), aspiration catheter (*n* = 5), and sputum (*n* = 5). Meanwhile, ten suspicious colonies were found and further identified by biochemical method, and finally five CR-KP isolates were cultured from radiant warmers or nurses’ hands during the peak of the outbreak.

**Table 1:** Characteristics of multi-drug resistance *K. pneumoniae*

Isolate No.	sex	Specimen	Date Of detection	STs	Carbapenemase	ESBL
TJ1	F	Sp	23/1/2015	ST11	KPC-2	CTX-14,TEM-1
TJ2	M	Bl	1/2/2015	ST888	NDM-1	CTX-15,TEM-1
TJ3	M	Ca	6/3/2015	ST11	KPC-2	CTX-14,TEM-1
TJ4	M	Bl	7/3/2015	ST11	KPC-2	CTX-15
TJ5	M	Sp	15/7/2015	ST11	KPC-2	CTX-14,TEM-1
TJ6	M	Sp	16/7/2015	ST11	KPC-2	CTX-14
TJ7	F	Ur	18/7/2015	ST20	NDM-1	TEM-1
TJ8	M	Ca	4/8/2015	ST11	KPC-2	CTX-14
TJ9	M	Ca	8/8/2015	ST11	KPC-2	CTX-14,TEM-1
TJ10	F	Bl	11/8/2015	ST11	KPC-2	CTX-14,TEM-1
TJ11	M	Ur	26/8/2015	ST11	KPC-2	CTX-14,TEM-1
TJ12	M	Bl	31/8/2015	ST11	KPC-2	CTX-15
TJ13	M	Bl	4/9/2015	ST11	KPC-2	CTX-14,TEM-1
TJ14	F	Ca	13/9/2015	ST20	NDM-1	CTX-14,TEM-1
TJ15	M	Sp	13/9/2015	ST11	KPC-2	CTX-14,TEM-1
TJ16	M	Ca	22/9/2015	ST20	NDM-1	CTX-14
TJ17	F	Sp	25/9/2015	ST11	KPC-2	CTX-14,TEM-1
TJ18	F	Ur	30/9/2015	ST11	KPC-2	CTX-14,TEM-1
TJ19	M	Bl	2/10/2015	ST11	KPC-2	CTX-14,TEM-1
TJ20	F	Ur	6/10/2015	ST11	KPC-2	CTX-14,TEM-1
TJ21	M	Ur	8/10/2015	ST11	KPC-2	CTX-14,TEM-1
TJ22	F	Ur	12/10/2015	ST11	KPC-2	CTX-14,TEM-1
TJ23	M	Bl	25/10/2015	ST11	KPC-2	–
TJ24	M	Bl	28/10/2015	ST20	NDM-1	CTX-14
TJ25	F	Ur	20/11/2015	ST11	KPC-2	CTX-14,TEM-1
TJ26	F	Bl	20/11/2015	ST11	KPC-2	CTX-14
TJ27	M	Ur	21/11/2015	ST11	KPC-2	CTX-14,TEM-1
TJ28^a^	–	Radiant warmer	8/9/2015	ST11	KPC-2	CTX-14,TEM-1
TJ29^a^	–	Radiant warmer	8/9/2015	ST20	NDM-1	CTX-14
TJ30^b^	–	Nurses hand	8/9/2015	ST11	KPC-2	CTX-14,TEM-1
TJ31^c^	–	Radiant warmer	8/9/2015	ST11	KPC-2	CTX-14,TEM-1
TJ32^b^	–	Nurses hand	8/9/2015	ST20	NDM-1	CTX-14

*F* female, *M* male, *Sp* sputum, *BL* blood, *Ur* urine, *Ca* aspiration catheter

^a^65 bed radiant warmer; ^b^nurses hand; ^c^55 bed radiant warmer

### Antimicrobial susceptibility patterns of *K. pneumoniae* isolates

The results of minimum inhibitory concentration (MIC) assays are shown in Table 2. All *K. pneumoniae* isolates were non-susceptible to ampicillin, cefoxitin, cefuroxime, cefotaxime, cefepime, ceftazidime, imipenem, meropenem, and piperacillin tazobactam, but were highly sensitive to tigecycline and colistin. The antibiotic resistance rates to gentamicin, amikacin, levofloxacin, ciprofloxacin, aztreonam, and trimethoprim-sulfamethoxazole were 78.1, 78.1, 78.1, 84.1, 78.1, and 21.9 %, respectively. Notably, strains producing NDM-1carbapenemase show different antibacterial susceptibility spectra from strains producing KPC-2 carbapenemase, including gentamicin, amikacin, levofloxacin, ciprofloxacin, aztreonam, and trimethoprim-sulfamethoxazole (see Table 2).

**Table 2:** Antibiotic susceptibility of *K. pneumoniae*

Minimal inhibitory concentration (ug/ml)		
Antibiotics	*K. pneumoniae*ST11 (*n* = 25)	NDM-1positive*K.pneumoniae* (*n* = 7)
	range	range
AMP	≥512	≥512
CAZ	≥512	≥512
CTX	128- > 512	64- > 512
FOX	64–256	64–256
FEP	64–256	16–64
CXM	≥512	≥512
ATM	≥512	1–2
GN	64–256	0.25–0.5
LEV	32–128	0.06–0.25
AK	≥512	1–2
CIP	≥32	0.06–1
TZP	64/4- > 256/4	128/4- > 256/4
SXT	1/19–2/38	≥256/4864
IPM	8–64	1–16
MEM	4–32	1–32
TGC	0.06–0.5	0.06–0.5
CO	0.03–0.5	0.06–0.25

*AMP* ampicillin (0.06–512), *CAZ* ceftazidime (0.06–512), *CTX* cefotaxime (0.06–512), *FEP* cefepime (0.06–512), *CXM* cefuroxime (0.06–512), *FOX* cefoxitin (0.06–512), *GN* gentamicin (0.06–512), *LEV* levofloxacin (0.06–512), *AK* amikacin (0.06–512), *CIP* ciprofloxacin (0.004-32), *ATM* Aztreonam (0.06–512), *IPM* imipenem (0.03–256), *MEM* meropenem (0.03–256), *TZP* piperacillin-tazobactam (0.25/4–256/4), *SXT* trimethoprim-sulfamethoxazole (0.25/4.75–256/4864), *TGC* tigecycline (0.03–256), *CO* colistin (0.03–256)

### Sequence typing and expression of drug-resistance genes

Based on the multi-locus sequence typing (MLST) results, the 27 isolates from unique patients were identified as Sequence Type (ST) 11 (ST11) (*n* = 22), ST20 (*n* = 4), and ST888 (*n* = 1), and the five environmental isolates were identified as ST11 (*n* = 3) and ST20 (*n* = 2). The percentages of isolates with carbapenemases KPC-2 and NDM-1were 81.5 % (22/27) and 18.5 % (5/27), respectively. All KPC-2-producing strains were ST11, and most of the NDM-1-positive strains were ST20 (*n* = 4). Three of the five environmental strains were KPC-2-positive *K. pneumoniae* ST11, and the other two environmental isolates were NDM-1-positive *K. pneumoniae* ST20. The rates of the ESBLs CTX-M-15, CTX-M-14, and TEM-1 were 9.4, 84.3, and 68.8 %, respectively. AmpC genes and other carbapenemases were not detected.

### The effect of infection control interventions

We monitored for neonatal infection with *K. pneumoniae* in future 4 months, and we detected multidrug-resistant *K. pneumoniae* in five patients, three patients, one patient and one patient, respectively, over these 4 months.

## Discussion

*K. pneumoniae* is an important nosocomial pathogen that can cause pneumonia, sepsis, and meningitis [15]. The emergence of CR-KP has created a challenge for treating *K. pneumoniae* infections, the mortality caused by CR-KP incection was high [16]. However, one infected neonate has died in our study. Mortality from *K. pneumoniae* infection is associated with a variety of factors, including underlying disease, nutritional status, and medical treatment, which may explain the discrepancy in mortality among different studies.

Underlying disease, low birth weight, antibacterial drug treatment, invasive procedures, and prolonged hospitalization have all been reported as extremely important risk factors of CRE infection [17, 18]. Of the 27 patients in this study, the percentages of subjects who had a low birth weight, who were treated with antibacterial drugs, and who underwent invasive procedures were 74.1 % (*n* = 20), 100 % (*n* = 27), and 77.8 % (*n* = 21) (data was not show), respectively. Additionally, we noticed that each nurse needed to take care of several infants and that each newborn was usually cared for by several nurses due to scheduling reasons. Overall, the newborns in our study possessed high risk factors for CRE infection, and this outbreak was likely spread by cross-infection.

As results showed, no significant differences in sex ratio and age between infection patients and non-infection patients. The CR-KP infection probably has no relation with age and gender in our study,which was similar with other study [19]. In addition, the average days for hospitalization in TJH is 11.8 days at present. However, the average hospital stay of the 27 patients including in this study was 29.9 days. Prolonged hospitalization may contribute to spreading of the ST11 and ST20 strains in neonatal ward.

The first KPC-2-producing *K. pneumoniae* isolate (TJ1) was isolated from a 23-day-old boy that was confirmed as infected with multidrug-resistant *K. pneumoniae* at another hospital. He was transferred to our hospital for treatment on January 23, 2015, and a sputum culture confirmed his infection with a multidrug-resistant *K. pneumoniae*. We learned that his mother had suffered from a respiratory tract infection for 2 weeks and 4 days. Through combination therapy with cefoperazone sulbactam, amoxicillin clavulanic acid, fluconazole, and meropenem, the child recovered and was discharged on February 5, 2015. Subsequently, KPC-2-positive strains with the same phenotype appeared in the same ward. All KPC-2-positive strains were detected in patients who had been hospitalized for more than 48 h, so we speculate that the origin of the KPC-2-positive *K. pneumoniae* ST11 was probably TJ1. Rodloff MD and his colleagues found that carriage of KPC-2-producing *K. pneumoniae* can last for 3 years [20]. It probably explain continuous outbreak after index case. Regrettably, the origin of the NDM-1-producing isolates in our study is still unknown.

The number of infection with *K. pneumoniae* decrease sharply in future 4 months. These declining numbers suggest that our efforts to control the continuous outbreak of multidrug-resistant *K. pneumoniae* were at least partially effective. However, there was one or two CR-KP detected after infection control interventions. It is difficult to eradicate the CR-KP by infection control measures [21]. Infection control measures were still need to improve, including routine screening of hospitalized patients, pathogens culture of medical equipment and screening the colonization status of contemporaneous patients.

This study has several limitations. Specifically, we only performed one environmental sampling and neglect screening of patients with perianal swabs to detect the rate of colonized patients. Additional samples might have provided stronger evidence to support our conclusion that the CR-KP was spread through cross-infection. Our data is also unable to confirm whether or not the CR-KP strains isolated in the later 3 months of our study are the same as the previous epidemic strain.

## Conclusions

In summary, we report that KPC-2-positive *K. pneumoniae* ST11 and NDM-1-positive *K. pneumoniae* ST20 are the two main clones that caused neonate infection in our hospital during the study period. During the outbreak, we successfully isolated these two main clones from a radiant warmer and from nurses’ hands. The radiant warmer and the nurses’ hands may have played an important role in harboring the *K. pneumoniae* producing KPC-2 or NDM-1 carbapenemases that caused persistent infection within the same ward. Following the emergence of CRE on a ward, Screening and infection control measures must be implemented to block nosocomial transmission. Active and effective infection control measures are indispensable for preventing a subsequent outbreak.

## Additional files

Additional file 1: Sequencing for resistance genes. (DOCX 17 kb)
Additional file 2: Sequencing for MLST. (DOCX 21 kb)
Additional file 3: Raw data of minimal inhibitory concentration. (XLSX 13 kb)

## Abbreviations

CRE: Carbapenem -resistant *Enterobacteriaceae*
CR-KP: Carbapenem resistance -*Klebsiella pneumoniae*
ESBLs: Extended-spectrum beta-lactamases
KPC-2: *K. pneumoniae* carbapenemase
MIC: Minimal inhibitory concentration
MLST: Multilocus sequence typing
NDM-1: New Delhi metallo-β-lactamase-1 enzymes
ST: Sequencing type
