# Supplementary material for: Nosocomial outbreak of KPC-2- and NDM-1-producing Klebsiella pneumoniae in a neonatal ward: a retrospective study
Source: BMC Infect Dis. 2016 Oct 12;16:563. doi: 10.1186/s12879-016-1870-y (PMC5062924; doi:10.1186/s12879-016-1870-y)
Supplement: Additional file 1: — Sequencing for resistance genes. (DOCX 17 kb) [file 12879_2016_1870_MOESM1_ESM.docx]

**sequencing for resistance gene**

CTX-14 GenBank accession number [KT459715.1](https://www.ncbi.nlm.nih.gov/nucleotide/1059525954?report=genbank&log$=nucltop&blast_rank=1&RID=XWR5U434016)

AGCGCGGCGCGTTGAGTACAGCGACAATACCGCCATGAACAAATTGATTGCCCAGCTCGGTGGCCCGGGAGGCGTGACGGCTTTTGCCCGCGCGATCGGCGATGAGACGTTTCGTCTGGATCGCACTGAACCTACGCTGAATACCGCCATTCCCGGCGACCCGAGAGACACCACCACGCCGCGGGCGATGGCGCAGACGTTGCGTCAGCTTACGCTGGGTCATGCGCTGGGCGAAACCCAGCGGGCGCAGTTGGTGACGTGGCTCAAAGGCAATACGACCGGCGCAGCCAGCATTCGGGCCGGCTTACCGACGTCGTGGACTGTGGGTGATAAGACCGGCAGCGGCGACTATGGCACCACAA

CTX-15 GenBank accession number [KT459659.1](https://www.ncbi.nlm.nih.gov/nucleotide/1059525842?report=genbank&log$=nucltop&blast_rank=13&RID=XWREAZ70014)

AGTCGAGTACGCAAACTTGCCGAATTAGAGCGGCAGTCGGGAGGCAGACTGGGTGTGGCATTGATTAACACAGCAGATAATTCGCAAATACTTTATCGTGCTGATGAGCGCTTTGCGATGTGCAGCACCAGTAAAGTGATGGCCGCGGCCGCGGTGCTGAAGAAAAGTGAAAGCGAACCGAATCTGTTAAATCAGCGAGTTGAGATCAAAAAATCTGACCTTGTTAACTATAATCCGATTGCGGAAAAGCACGTCAATGGGACGATGTCACTGGCTGAGCTTAGCGCGGCCGCGCTACAGTACAGCGATAACGTGGCGATGAATAAGCTGATTGCTCACGTTGGCGGCCCGGCTAGCGTCACCGCGTTCGCCCGACAGCTGGGAGACGAAACGTTCCGTCTCGACCGTACCGAGCCGACGTTAAACACCGCCATTCCGGGCGATCCGCGTGATACCACTTCACCTCGGGCAATGGCGCAAACTCTGCGGAATCTGACGCTGGGTAAAGCATTGGGCGACAGCCAACGGGCGCAGCTGGTGACATGGATGAAAGGCAATACCACCGGTGCAGCGAGCATTCAGGCTGGACTGCCTGCTTCCTGGGTTGTGGGGGATAAAACCGGCAGCGGTGGCTATGGCACCACCCCCAAAATTCG

TEM-1 GenBank accession number [KP634895.1](https://www.ncbi.nlm.nih.gov/nucleotide/814603645?report=genbank&log$=nucltop&blast_rank=1&RID=XWRP9RDZ014)

GCTATCATTTTTGCGGCTTTTGCCTTCCTGTTTTTGCTCACCCAGAAACGCTGGTGAAAGTAAAAGATGCTGAAGATCAGTTGGGTGCACGAGTGGGTTACATCGAACTGGATCTCAACAGCGGTAAGATCCTTGAGAGTTTTCGCCCCGAAGAACGTTTTCCAATGATGAGCACTTTTAAAGTTCTGCTATGTGGTGCGGTATTATCCCGTGTTGACGCCGGGCAAGAGCAACTCGGTCGCCGCATACACTATTCTCAGAATGACTTGGTTGAGTACTCACCAGTCACAGAAAAGCATCTTACGGATGGCATGACAGTAAGAGAATTATGCAGTGCTGCCATAACCATGAGTGATAACACTGCTGCCAACTTACTTCTGACAACGATCGGAGGACCGAAGGAGCTAACCGCTTTTTTGCACAACATGGGGGATCATGTAACTCGCCTTGATCGTTGGGAACCGGAGCTGAATGAAGCCATACCAAACGACGAGCGTGACACCACGATGCCTGCAGCAATGGCAACAACGTTGCGCAAACTATTAACTGGCGAACTACTTACTCTAGCTTCCCGGCAACAATTAATAGACTGGATGGAGGCGGATAAAGTTGCAGGACCACTTCTGCGCTCGGCCCTTCCGGCTGGCTGGTTTATTGCTGATAAATCTGGAGCCGGTGAGCGTGGGTCTCGCGGTATCATTGCAGCACTGGGGCCAGATGGTAAGCCCTCCCGTATCGTAGTTATCTACACGACGGGGAGTCAGGCAACTATGGATGAACGAAATAGACAGATCGCTGAGATAGGTGCCTCACTGATAAGTGTGGGAAATAGG

KPC-2 GenBank accession number [KT001101.1](https://www.ncbi.nlm.nih.gov/nucleotide/870898370?report=genbank&log$=nucltop&blast_rank=48&RID=XWRTSVE101R)

ATACTGTCTCTCTGGCCGCTGGCTGGCTTTTCTGCCACCGCGCTGACCAACCTCGTCGCGGAACCATTCGCTAAACTCGAACAGGACTTTGGCGGCTCCATCGGTGTGTACGCGATGGATACCGGCTCAGGCGCAACTGTAAGTTACCGCGCTGAGGAGCGCTTCCCACTGTGCAGCTCATTCAAGGGCTTTCTTGCTGCCGCTGTGCTGGCTCGCAGCCAGCAGCAGGCCGGCTTGCTGGACACACCCATCCGTTACGGCAAAAATGCGCTGGTTCCGTGGTCACCCATCTCGGAAAAATATCTGACAACAGGCATGACGGTGGCGGAGCTGTCCGCGGCCGCCGTGCAATACAGTGATAACGCCGCCGCCAATTTGTTGCTGAAGGAGTTGGGCGGCCCGGCCGGGCTGACGGCCTTCATGCGCTCTATCGGCGATACCACGTTCCGTCTGGACCGCTGGGAGCTGGAGCTGAACTCCGCCATCCCAGGCGATGCGCGCGATACCTCATCGCCGCGCGCCGTGACGGAAAGCTTACAAAAACTGACACTGGGCTCTGCACTGGCTGCGCCGCAGCGGCAGCAGTTTGTTGATTGGCTAAAGGGAAACACGACCGGCAACCACCGCATCCGCGCGGCGGTGCCGGCAGACTGGGCAGTCGGAGACAAAACCGGAACCTGCGGAGTGTATGGCACGGCAAATGACTATGCCGTCGTCTGGCCCACTGGGCGCGCACCTATTGTGTTGGCCGTCTACACCCGGGCGCCTAACAAGGATGACAAGCACAGCGAGGCCGTCATCGCCGCTGCGGCTAGACTCGCGCTCGAGGGATTGGGCGTCAACGGCATAGGGGCCTTTAAAAAAAAAAGGGGGTGGGTT

[NDM-1 GenBank accession number HQ662553.1](https://www.ncbi.nlm.nih.gov/nucleotide/354589535?report=genbank&log$=nucltop&blast_rank=1&RID=XWRWPYNP014)

GGTAGCGCGTCATACCGCCATCTTGTCCTGATGCGCGTGAGTCACCACCGCCAGCGCGACCGGCAGGTTGATCTCCTGCTTGATCCAGTTGAGGATCTGGGCGGTCTGGTCATCGGTCCAGGCGGTATCGACCACCAGCACGCGGCCGCCATCCCTGACGATCAAACCGTTGGAAGA
