# Supplementary material for: Nosocomial outbreak of KPC-2- and NDM-1-producing Klebsiella pneumoniae in a neonatal ward: a retrospective study
Source: BMC Infect Dis. 2016 Oct 12;16:563. doi: 10.1186/s12879-016-1870-y (PMC5062924; doi:10.1186/s12879-016-1870-y)
Supplement: Additional file 2: — Sequencing for MLST. (DOCX 21 kb) [file 12879_2016_1870_MOESM2_ESM.docx]

**sequencing for MLST**

**Kpn MLST11**

**rpoB**

GGCTGGTACGTGTAGAGCGTGCGGTGAAGAGCGTCTGTCTCTTGGCGATCTGGATACCCTGATGCCTCAGGATATGATCAACGCCAAGCCGATTTCCGCAGCAGTGAAAGAGTTCTTTGGTTCCAGCCAGCTGTCTCAGTTTATGGACCAGAACAACCCGCTGTCTGAGATTACGCACAAACGTCGTATCTCCGCACTCGGCCCAGGCGGTCTGACCCGTGAGCGCGCAGGCTTCGAAGTTCGAGACGTACACCCGACCCACTACGGTCGCGTATGTCCGATCGAAACGCCTGAAGGTCCGAACATCGGTCTGATTAACTCCCTGTCCGTGTACGCGCAGACCAACGAATATGGCTTCCTTGAGACGCCGTATCGTAAAGTGACCAACGGTGTGGTTACTGACGAAATTCACTACCTGTCTGCTATCGAAGAAGGCAACTACGTTATCGCTCAGGCGAACTCCAACCTGGATGAAAACGGCCACTTCGTAGAAGATCTGGTTACCTGCCGTAGCAAAGGCGAATCCAGCTTGTTCAGCCGCGACCAGGTTGACTACATGGACGTATCCACCCAGCAGGTGGTATCCGTCGGTGCGTCCCTGATCCCGTTCCTGGAACACGATGACGCCAACCGTGCATTGATGGGTGCGAACATGCAACGTCAGGCGGTTCCGACTCTGCGCGCTGATAAGCCGCTGGTTGGTACCGGTATGGAACGTGCTGTTGCCGTTGACTCCGGTGTTACTGCCGTGGCTAAACGTGGCGGTACCGTTCAGTACGTGGATGCTTCCCGTATCGTTATCAAAGTTAACGAAGACGAAATGTACCCGGGCGAAGCAGGTATCGACATCTATAACCTGACCAAGTACACCCGTTCTAACCAGAACACCTGCATCAACCAGATGCCTTGCGTGTCCCTGGGCGAACCTATTGAGCGCGGCGACGTGCTGGCAAACGGCCCGTCACCGACCCCGGGAACTGGCCCTGGGCCAAACATGCTG

**gapA**

CACGGAACGTTGAGTGAGACGGTCATCTGGTCGTTAACGGTAAAAAAATCCGTGTTACCGCTGAACGTGACCCGGCTAACCTGAAGTGGGACGAAGTTGGTGTTGACGTTGTTGCTGAAGCAACCGGTATCTTCCTGACCGACGAAACCGCTCGTAAACACATCACCGCTGGCGCGAAAAAAGTCGTTCTGACTGGCCCGTCCAAAGACAACACTCCGATGTTCGTTCGCGGCGCTAACTTCGACGCTTACGCTGGCCAGGACATCGTTTCCAACGCTTCCTGCACCACTAACTGCCTGGCGCCGCTGGCTAAAGTTATCAACGACAACTTCGGTATCGTTGAAGGCCTGATGACCACCGTCCACGCTACCACCGCTACTCAGAAAACCGTTGATGGCCCGTCTCACAAAGACTGGCGCGGCGGCCGCGGCGCAGCTCAGAACATCATCCCGTCCTCTACCGGCGCTGCTAAAGCAGTAGGTAAAGTACTGCCAGAACTGAACGGCAAACTGACCGGTATGGCGTTCCGCGTTCCAACTCCGAACGTATCTGTTGTTGACCTGACCGTTCGTCTGGAAAAAGCAGCGTCCTACGAAGAAATCAAGAAAGCCATCAAAGCGCCTTTCCGAAGGAA

**mdh**

TGGACGACTCGCTCGGTTACGCCGGGCGTGGCGGTAGATCTAAGTCATATCCCCACAGATGTAAAAATTAAAGGATTTTCCGGTGAAGACGCTACTCCGGCGCTGGAAGGCGCGGATGTAGTGCTGATCTCCGCGGGCGTGGCGCGTAAGCCCGGCATGGATCGTTCCGACCTGTTTAATGTGAATGCGGGTATCGTGAAGAACCTCGTGCAGCAGATTGCCAAAACCTGCCCGCAGGCCTGCATCGGCATTATCACCAACCCGGTGAATACCACCGTGGCTATCGCCGCCGAAGTACTGAAAAAAGCCGGCGTGTACGATAAAAACAAACTGTTCGGCGTTACCACGCTGGACATCATCCGTTCCAATACCTTTGTGGCGGAGCTGAAAGGTAAATCGGCAACCGAGGTGGAAGTCCCGGTCATTGGTGGTCACTCCGGGGTCACCATTCTGCCTTTACTGTCGCAGATCCCCGGCGTCAGCTTTAGCGATCAGGAAATTGCCGACCTGACTAAACGTATTCAGAACGCCGGTACCGAAGTCGTGGAAGCGAAAGCGGGCGGCGGGTCGGCGACCTTGTCGATGGGCCAGGCGGCTGCCCGTTTTGGTCTCTCTCTGGTTCGCGCCATGCAGGGGGAAAAAGGCGTGGTGGAGTGCGCCTACGTGGAAGGCGACGGCCACTATGCGCGTTTCTTCTCCCAGCCGCTGCGTGGGGGAAAAAAAAGGGGGAAAAA

**pgi**

GCTTCTCGGTGCGGACCGAAGCGATTCTGCCGTACGACCAGTACATGCACCGCTTTGCCGCTTACTTCCAGCAGGGCAACATGGAGTCCAACGGTAAGTATGTTGACCGTAACGGCCACGCGGTAGACTACCAGACTGGCCCAATCATCTGGGGTGAGCCGGGCACCAACGGTCAGCACGCGTTCTACCAGCTGATCCACCAGGGCACCAAAATGGTACCGTGCGATTTCATCGCTCCGGCTATCACCCACAACCCGCTGTCTGACCACCATCAGAAACTGCTGTCTAACTTCTTCGCCCAGACCGAGGCCCTGGCCTTTGGTAAATCCCGCGAAGTGGTTGAGCAGGAATATCGCGATCAGGGTAAAGACCCGGCGACCCTGGAGCACGTGGTGCCGTTCAAAGTGTTCGAAGGTAACCGCCCGACTAACTCCATCCTGCTGCGTGAGATCACCCCGTTCAGCCTCGGGGCGCTGATTGCCCTGTACGAGCACAAAATCTTCACCCAGGGCGCGATCCTCAACATCTTCACCTTTGACCAGTGGGGCGTTGAGCTGGGCAAACAGCTGGCTAACCGCATCCTGCCGGAGCTGAAAGACGGCAGCGAAGTTAGCAGCCACGACAGCTCTACTAACGGCTTAAACCCCCCCCCAAAAAAAAAAAAAACCAAAAGC

**phoE**

ACGGGATGTCGTCTCGTTGATGCCGAGTTTGTTATCGCTTTTCAGCTGGTTGATTTTGTAATCCACGAAGGCGTTCATGTTTTTGTTGAAGTAGTAGGTCAGGCCCACGTCGATGTAGTTAACCAGATCTTCACTCCCCACCCCTTCGATATCCTTCCCTTTCGACAGCACATAGCCGAGGGACGGACGCAGACCGAAGTCGAACTGATACTGCGCCACCGCTTCAAAGTTCTGCGCTTTGTTGGCAAAGCCGCCGCTGATCGGGGTCATCTTGCGGGTTTCAGAGTACATGGTCGCCAGGTAGATATTGTTGGCGTCATATTTCAGGCCGGTCGCCCAGGCTTCCGCTTTCGAACCCTGGCCGCGGGCCAGCAGGTTCTGATCGTTGGTACGGTCGGAGCTGGTGTAGGCTGCGCTGACGGCGAAGTCGCTGCCGCCGAAATCATAGCTTAACGAGGTGCCGACGCCGTCGCCGTTCTGTTTCTTCGCTTCACGGCCTTCGTTTTTACCCTGGTACTGCAGGGTCAGATCCAGGCCATCCACCAGACCGAAGAAGTCGGTGTGCCGGGTAAGGAAAA

**infB**

TCTGGTACCGCCTCGGTGCTTCACGTCGAAACCGACAACGGCATGATCACCTTCCTGGATACCCCGGGCCACGCCGCGTTTACCTCCATGCGTGCTCGTGGCGCGCAGGCGACGGATATCGTGGTTCTGGTGGTGGCGGCAGACGACGGCGTGATGCCGCAGACTATCGAAGCTATCCAGCACGCTAAAGCGGCGCAGGTACCGGTGGTAGTGGCGGTGAACAAGATCGATAAGCCAGAAGCCGATCCGGATCGCGTGAAGAACGAACTGTCCCAGTACGGCATCCTGCCGGAAGAGTGGGGCGGCGAGAGCCAGTTTGTCCACGTTTCCGCGAAAGCGGGTACCGGCATCGACGACCTGCTGGACGCGATCCTGCTGCAGGCTGAAGTTCTG

**tonB**
TGACGTCTCGACGCGCCGATAGAGATCACAATGGTGGCGCCGGCCGATCTTGAGCCGCCTCCGGCGGCGCAGCCTGTCGTGGAGCCCGTTGTTGAACCCGAACCTGAGCCGGAGCCAGAGGTAGCGCCTGAACCGCCGAAAGAGGCGCCGGTGGTGATCCATAAACCGGAACCTAAGCCGAAGCCCAAACCTAAACCCAAGCCTAAGCCGGAGAAAAAGGTTGAACAGCCGAAGCGGGAAGTGAAGCCGGCAGCAGAGCCGCGTCCGGCCTCGCCGTTTGAAAACAACAATACGGCGCCGGCGCGTACAGCGCCAAGTACCTCGACCGCAGCGGCTAAACCCACCGTTACTGCTCCGAGCGGCCCGCGGGCGATCAGCCGCGTTCAGCCGTCCTATCCGCCGCGCGCTCAGGCGCTGCGCATTGAAGGGACGGTACGGGTGAAGTTTGACGTTTCGCCTGATGGCCGCATTGATAATCTGCAGATCCTCTCTGCTAGCCGGGGCGAATAAAACGCGGGCCCC

**Kpn MLST20**

**rpoB**

GGTGGGCGTCGTGTAGAGCGTGCGGTGAAGAGCGTCTGTCTCTTGGCGATCTGGATACCCTGATGCCTCAGGATATGATCAACGCCAAGCCGATTTCCGCAGCAGTGAAAGAGTTCTTTGGTTCCAGCCAGCTGTCTCAGTTTATGGACCAGAACAACCCGCTGTCTGAGATTACGCACAAACGTCGTATCTCCGCACTCGGCCCAGGCGGTCTGACCCGTGAGCGCGCAGGCTTCGAAGTTCGAGACGTACACCCGACCCACTACGGTCGCGTATGTCCGATCGAAACGCCTGAAGGTCCGAACATCGGTCTGATTAACTCCCTGTCCGTGTACGCGCAGACCAACGAATATGGCTTCCTTGAGACGCCGTATCGTAAAGTGACCGACGGTGTGGTTACTGACGAAATTCACTACCTGTCTGCTATCGAAGAAGGCAACTACGTTATCGCTCAGGCGAACTCCAACCTGGATGAAAACGGCCACTTCGTAGAAGATCTGGTTACCTGCCGTAGCAAAGGCGAATCCAGCTTGTTCAGCCGCGACCAGGTTGACTACATGGACGTATCCACCCAGCAGGTGGTATCCGTCGGTGCGTCCCTGATCCCGTTCCTGGAACACGATGACGCCAACCGTGCATTGATGGGTGCGAACATGCAACGTCAGGCGGTTCCGACTCTGCGCGCTGATAAGCCGCTGGTTGGTACCGGTATGGAACGTGCTGTTGCCGTTGACTCCGGTGTTACTGCCGTGGCTAAACGTGGCGGTACCGTTCAGTACGTGGATGCTTCCCGTATCGTTATCAAAGTTAACGAAGACGAGATGTACCCGGGCGAAGCAGGTATCGACATCTATAACCTGACCAAGTACACCCGTTCTAACCAGAACACCTGCATCAACCAGATGCCTTGCGTGTCCTTGGGCGAACCTATTGAGCGCGGCGACGTGCTGGCAAACGGCCCGTCCACCGACCTCGGTGAACTGGCGCTGGGTCAAACATGCGTGTAGC

**gapA**

AACGATCGTTGAGTGAGACGGTCATCTGGTCGTTAACGGTAAAAAAATCCGTGTTACCGCTGAACGTGACCCGGCTAACCTGAAGTGGGACGAAGTTGGTGTTGACGTTGTTGCTGAAGCAACCGGTATCTTCCTGACCGACGAAACCGCTCGTAAACACATCACCGCTGGCGCGAAAAAAGTCGTTCTGACTGGCCCGTCCAAAGACAACACTCCGATGTTCGTTCGCGGCGCTAACTTCGACGCTTACGCTGGCCAGGACATCGTTTCCAACGCTTCCTGCACCACCAACTGCCTGGCGCCGCTGGCTAAAGTTATCAACGACAACTTCGGTATCGTTGAAGGCCTGATGACCACCGTCCACGCTACCACCGCTACTCAGAAAACCGTTGATGGCCCGTCTCACAAAGACTGGCGCGGCGGCCGCGGCGCAGCTCAGAACATCATCCCGTCCTCTACCGGCGCTGCTAAAGCAGTAGGTAAAGTACTGCCAGAACTGAACGGCAAACTGACCGGTATGGCGTTCCGCGTTCCAACTCCGAACGTATCTGTTGTTGACCTGACCGTTCGTCTGGAAAAAGCAGCGTCCTACGAAGAAATCAAGAAAGCCATCAAAGCCGCTTTCTGAAGAAATACTCAA

**pgi**

GCTTCATCGGTGCGGAACCGAAGCGATTCTGCCGTACGACCAGTACATGCACCGCTTTGCCGCTTACTTCCAGCAGGGCAACATGGAGTCCAACGGTAAGTATGTTGACCGTAACGGCCACGCGGTAGACTACCAGACTGGCCCAATCATCTGGGGTGAGCCGGGCACCAACGGTCAGCACGCGTTCTACCAGCTGATCCACCAGGGCACCAAAATGGTACCGTGCGATTTCATCGCTCCGGCTATCACCCACAACCCGCTGTCTGACCACCATCAGAAACTGCTGTCTAACTTCTTCGCCCAGACCGAGGCCCTGGCCTTTGGTAAATCCCGCGAAGTGGTTGAGCAGGAATATCGCGATCAGGGTAAAGACCCGGCGACCCTGGAGCACGTGGTGCCGTTCAAAGTGTTCGAAGGTAACCGCCCGACTAACTCCATCCTGCTGCGTGAGATCACCCCGTTCAGCCTCGGGGCGCTGATTGCCCTGTACGAGCACAAAATCTTCACCCAGGGCGCGATCCTCAACATCTTCACCTTTGACCAGTGGGGCGTTGAGCTGGGCAAACAGCTGGCTAACCGCATCCTGCCGGAGCTGAAAGACGGCAGCGAAGTTAGCAGCCACGACAGCTCTACTAACGGCCTGATACCCCCCTATAAAACCCCC

**mdh**

TGGACGACTCGCTCGGTTACGCCGGGCGTGGCGGTAGATCTAAGTCATATCCCCACAGATGTAAAAATTAAAGGATTTTCCGGTGAAGACGCTACTCCGGCGCTGGAAGGCGCGGATGTAGTGCTGATCTCCGCGGGCGTGGCGCGTAAGCCCGGCATGGATCGTTCCGACCTGTTTAATGTGAATGCGGGTATCGTGAAGAACCTCGTGCAGCAGATTGCCAAAACCTGCCCGCAGGCCTGCATCGGCATTATCACCAACCCGGTGAATACCACCGTGGCTATCGCCGCCGAAGTACTGAAAAAAGCCGGCGTGTACGATAAAAACAAACTGTTCGGCGTTACCACGCTGGACATCATCCGTTCCAATACCTTTGTGGCGGAGCTGAAAGGTAAATCGGCAACCGAGGTGGAAGTCCCGGTCATTGGTGGTCACTCCGGGGTCACCATTCTGCCTTTACTGTCGCAGATCCCCGGCGTCAGCTTTAGCGATCAGGAAATTGCCGACCTGACTAAACGTATTCAGAACGCCGGTACCGAAGTCGTGGAAGCGAAAGCGGGCGGCGGGTCGGCGACCTTGTCGATGGGCCAGGCGGCTGCCCGTTTTGGTCTCTCTCTGGTTCGCGCCATGCAGGGGGAAAAAGGCGTGGTGGAGTGCGCCTACGTGGAAGGCGACGGCCACTATGCGCGTTTCTTCTCCCAGCCGCTGCGTGGGGGAAAAAAAAGGGGGAAAAA

**phoE**

CTGCGCTGGACTGGATCTGACCCTGCAGTACCAGGGTAAAAACGAAGGCCGTGAAGCGAAGAAACAGAACGGCGACGGCGTCGGCACCTCGTTAAGCTATGATTTCGGCGGCAGCGACTTCGCCGTCAGCGCAGCCTACACCAGCTCCGACCGTACCAACGATCAGAACCTGCTGGCCCGCGGCCAGGGTTCGAAAGCGGAAGCCTGGGCGACCGGCCTGAAATATGACGCCAACAATATCTACCTGGCGACCATGTACTCTGAAACCCGCAAGATGACCCCGATCAGCGGCGGCTTTGCCAACAAAGCGCAGAACTTTGAAGCGGTGGCGCAGTATCAGTTCGACTTCGGTCTGCGTCCGTCCCTCGGCTATGTGCTGTCGAAAGGGAAGGATATCGAAGGGGTGGGAAGTGAGGATCTGGTTAACTACATTGACGTGGGCCTGACCTACTACTTCAACAAAAACATGAACGCCTTCGTGGATTACAAAATCAACCAGCTGAAAAGCGATAACAAACTCGGCATCAACGATGACGACATCGTCGCGCTGGGTATCACCTACAGTTTCTTGAACAAAA

**infB**

AGGAACAGCCTCGGTGCTTCCACGTCGAAACCGACAACGGCATGATCACCTTCCTGGATACCCCGGGCCACGCCGCGTTTACCTCCATGCGTGCTCGTGGCGCGCAGGCGACGGATATCGTGGTTCTGGTGGTGGCGGCAGACGACGGCGTGATGCCGCAGACTATCGAAGCTATCCAGCACGCTAAAGCGGCGCAGGTACCGGTGGTAGTGGCGGTGAACAAGATCGATAAGCCAGAAGCCGATCCGGATCGCGTGAAGAACGAACTGTCCCAGTACGGCATCCTGCCGGAAGAGTGGGGCGGCGAGAGCCAGTTTGTCCACGTTTCCGCGAAAGCGGGTACCGGCATCGACGACCTGCTGGACGCGATCCTGCTGCAGGCTGAAGTTCTTGAGCTGAAAGCGA

**tonB**
TGACGTCTCGACGCGCCGATAGAGATCACAATGGTGGCGCCGGCCGATCTTGAGCCGCCTCCGGCGGCGCAGCCTGTCGTGGAGCCCGTTGTTGAACCCGAACCTGAGCCGGAGCCAGAGGTAGCGCCTGAACCGCCGAAAGAGGCGCCGGTGGTGATCCATAAACCGGAACCTAAGCCGAAGCCCAAACCTAAACCCAAGCCTAAGCCGGAGAAAAAGGTTGAACAGCCGAAGCGGGAAGTGAAGCCGGCAGCAGAGCCGCGTCCGGCCTCGCCGTTTGAAAACAACAATACGGCGCCGGCGCGTACAGCGCCAAGTACCTCGACCGCAGCGGCTAAACCCACCGTTACTGCTCCGAGCGGCCCGCGGGCGATCAGCCGCGTTCAGCCGTCCTATCCGCCGCGCGCTCAGGCGCTGCGCATTGAAGGGACGGTACGGGTGAAGTTTGACGTTTCGCCTGATGGCCGCATTGATAATCTGCAGATCCTCTCTGCTAGCCGGGGCGAATAAAACGCGGGCCCC

**Kpn MLST888**

**rpoB**

GGTGCCTGGTCGTGTAGAGCGTGCGGTGACGAGCGTCTGTCTCTTGGCGATCTGGATACCCTGATGCCTCAGGATATGATCAACGCCAAGCCGATTTCCGCAGCAGTGAAAGAGTTCTTTGGTTCCAGCCAGCTGTCTCAGTTTATGGACCAGAACAACCCGCTGTCTGAGATTACGCACAAACGTCGTATCTCCGCACTCGGCCCAGGCGGTCTGACCCGTGAGCGCGCAGGCTTCGAAGTTCGAGACGTACACCCGACCCACTACGGTCGCGTATGTCCGATCGAAACGCCTGAAGGTCCGAACATCGGTCTGATTAACTCCCTGTCCGTGTACGCGCAGACCAACGAATATGGCTTCCTTGAGACGCCGTATCGTAAAGTGACCAACGGTGTGGTTACTGACGAAATTCACTACCTGTCTGCTATCGAAGAAGGCAACTACGTTATCGCTCAGGCGAACTCCAACCTGGATGAAAACGGCCACTTCGTAGAAGATCTGGTTACCTGCCGTAGCAAAGGCGAATCCAGCTTGTTCAGCCGCGACCAGGTTGACTACATGGACGTATCCACCCAGCAGGTGGTATCCGTCGGTGCGTCCCTGATCCCGTTCCTGGAACACGATGACGCCAACCGTGCATTGATGGGTGCGAACATGCAACGTCAGGCGGTTCCGACTCTGCGCGCTGATAAGCCGCTGGTTGGTACCGGTATGGAACGTGCTGTTGCCGTTGACTCCGGTGTTACTGCCGTGGCTAAACGTGGCGGTACCGTTCAGTACGTGGATGCTTCCCGTATCGTTATCAAAGTTAACGAAGACGAGATGTACCCGGGCGAAGCAGGTATCGACATCTATAACCTGACCAAGTACACCCGTTCTAACCAGAACACCTGCATCAACCAGATGCCTTGCGTGTCCCTGGGCGAACCTATTGAGCGCGGCGACGTGCTGGCAAACGGCCCGTCCACCGACCTCGGTGAACTGGCGCTGGGTCAAACATGCGTGTAACGTTATGCCTGGACG

**gapA**

TCACGTCGTTGAGTGAAGACGGTCATCTGGTCGTTAACGGTAAAAAAATCCGTGTTACCGCTGAACGTGACCCGGCTAACCTGAAGTGGGACGAAGTTGGTGTTGACGTTGTTGCTGAAGCAACCGGTATCTTCCTGACCGACGAAACCGCTCGTAAACACATCACCGCTGGCGCGAAAAAAGTCGTTCTGACTGGCCCGTCCAAAGACAACACTCCGATGTTCGTTCGCGGCGCTAACTTCGACGCTTACGCTGGCCAGGACATCGTTTCCAACGCTTCCTGCACCACCAACTGCCTGGCGCCGCTGGCTAAAGTTATCAACGACAACTTCGGTATCGTTGAAGGCCTGATGACCACCGTCCACGCTACCACCGCTACTCAGAAAACCGTTGATGGCCCGTCTCACAAAGACTGGCGCGGCGGCCGCGGCGCAGCTCAGAACATCATCCCGTCCTCTACCGGCGCTGCTAAAGCAGTAGGTAAAGTACTGCCAGAACTGAACGGCAAACTGACCGGTATGGCGTTCCGCGTTCCGACTCCGAACGTATCTGTTGTTGACCTGACCGTTCGTCTGGAAAAAGCAGCGTCCTACGAAGAAATCAAGAAAGCCATCAAGCGCTTTTTTTAAAA

**mdh**

AAGTATGACTCGCTCGGTTACGCCGGGCGTGGCGGTAGATCTAAGTCATATCCCCACAGATGTAAAAATTAAAGGATTTTCCGGTGAAGACGCTACTCCGGCGCTGGAAGGCGCGGATGTAGTGCTGATCTCCGCGGGCGTGGCGCGTAAGCCCGGCATGGATCGTTCCGACCTGTTTAATGTGAATGCGGGTATCGTGAAGAACCTCGTGCAGCAGATTGCCAAAACCTGCCCGCAGGCCTGCATCGGCATTATCACCAACCCGGTGAATACCACCGTGGCTATCGCCGCCGAAGTACTGAAAAAAGCCGGCGTGTACGATAAAAACAAACTGTTCGGCGTTACCACGCTGGACATCATCCGTTCCAATACCTTTGTGGCGGAGCTGAAAGGTAAATCGGCAACCGAGGTGGAAGTCCCGGTCATTGGTGGTCACTCCGGGGTCACCATTCTGCCTTTACTGTCGCAGATCCCCGGCGTCAGCTTTAGCGATCAGGAAATTGCCGACCTGACTAAACGTATTCAGAACGCCGGTACCGAAGTCGTGGAAGCGAAAGCGGGCGGCGGGTCGGCGACCTTGTCGATGGGCCAGGCGGCTGCCCGTTTTGGTCTCTCTCTGGTTCGCGCCATGCAGGGGGAAAAAGGCGTGGTGGAGTGCGCCTACGTGGAAGGCGACGGCCACTATGCGCGTTTCTTCTCCCAGCCGCTGCTGTGGGGAAAAAAACGGGACCTGGG

**phoE**

CTGGCTGATGGCTGGATCTGACCCTGCAGTACCAGGGTAAAAACGAAGGCCGTGAAGCGAAGAAACAGAACGGCGACGGCGTCGGCACCTCGTTAAGCTATGATTTCGGCGGCAGCGACTTCGCCGTCAGCGCAGCCTACACCAGCTCCGACCGTACCAACGATCAGAACCTGCTGGCCCGCGGCCAGGGTTCGAAAGCGGAAGCCTGGGCGACCGGCCTGAAATATGACGCCAACAATATCTACCTGGCGACCATGTACTCTGAAACCCGCAAGATGACCCCGATCAGCGGCGGCTTTGCCAACAAAGCGCAGAACTTTGAAGCGGTGGCGCAGTATCAGTTCGACTTCGGTCTGCGTCCGTCCCTCGGCTATGTGCTGTCGAAAGGGAAGGATATCGAAGGGGTGGGAAGTGAGGATCTGGTTAACTACATTGACGTGGGCCTGACCTACTACTTCAACAAAAACATGAACGCCTTCGTGGATTACAAAATCAACCAGCTGAAAAGCGATAACAAACTCGGCATCAACGATGACGACATCGTCGCGCTGGGTATCACTACAGTTTCCTGATCAAAACAC

**infB**

CTGCGGAACAGCCTCGGTGCTTCCACGTCGAAACCGACAACGGCATGATCACCTTCCTGGATACCCCGGGCCACGCCGCGTTTACCTCCATGCGTGCTCGTGGCGCGCAGGCGACGGATATCGTGGTTCTGGTGGTGGCGGCAGACGACGGCGTGATGCCGCAGACTATCGAAGCTATCCAGCACGCTAAAGCGGCGCAGGTACCGGTGGTAGTGGCGGTGAACAAGATCGATAAGCCAGAAGCCGATCCGGATCGCGTGAAGAACGAACTGTCCCAGTACGGCATCCTGCCGGAAGAGTGGGGCGGCGAGAGCCAGTTCGTCCACGTTTCCGCGAAAGCCGGTACCGGCATCGACGACCTGCTGGACGCGATCCTGCTGCAGGCTGAAGTTCTTG

**tonB**

ACGTCGTCTCGACGCGCCGATAGAGATCACAATGGTGGCGCCGGCCGATCTTGAGCCGCCTCCGGCGGCGCAGCCTGTCGTGGAGCCCGTTGTTGAACCCGAACCTGAGCCGGAGCCAGAGGTAGCGCCTGAACCGCCGAAAGAGGCGCCGGTGGTGATCCATAAACCGGAACCTAAGCCGAAGCCCAAACCTAAACCCAAGCCTAAGCCGGAGAAAAAGGTTGAACAGCCGAAGCGGGAAGTGAAGCCGGCAGCAGAGCCGCGTCCGGCCTCGCCGTTTGAAAACAACAATACGGCGCCGGCGCGTACAGCGCCAAGTACCTCGACCGCAGCGGCTAAACCCACCGTTACTGCTCCGAGCGGCCCGCGGGCGATCAGCCGCGTTCAGCCGTCCTATCCGCCGCGCGCTCAGGCGCTGCGCATTGAAGGGACGGTACGGGTGAAGTTTGACGTTTCGCCTGATGGCCGCATTGATAATCTGCAGATCCTCTCTGCTCACCCGGCGAATAAAAAAGCCGAAACA
